# Lipid Fraction from *Agaricus brasiliensis* as a Potential Therapeutic Agent for Lethal Sepsis in Mice

**DOI:** 10.3390/antiox13080927

**Published:** 2024-07-30

**Authors:** Kely Campos Navegantes Lima, Silvia Leticia de França Gaspar, Ana Ligia de Brito Oliveira, Sávio Monteiro dos Santos, Lucas Benedito Gonçalves Quadros, Juliana Pinheiro de Oliveira, Rayane Caroline dos Santos Pereira, Alexandre Guilherme da Silva Dias, Lucas da Silva Gato, Leonardo Yuji Nihira Alencar, Alanna Lorena Pimentel dos Santos, Gilson Pires Dorneles, Pedro Roosevelt Torres Romão, Herta Stutz, Vanessa Sovrani, Marta Chagas Monteiro

**Affiliations:** 1 Neuroscience and Cellular Biology Post Graduation Program, Institute of Biological Sciences, Federal University of Pará, Pará 66075-110, Brazil; profkelynavelima@gmail.com (K.C.N.L.); farmanaligia@gmail.com (A.L.d.B.O.); 2 School of Pharmacy, Health Science Institute, Federal University of Pará, Belém 66075-110, Brazil; leticiagaspar.farma@hotmail.com (S.L.d.F.G.); lucasquadros8@gmail.com (L.B.G.Q.); julianaoliveira.p98@gmail.com (J.P.d.O.); rayane.pereira@ics.ufpa.br (R.C.d.S.P.); alexandre.dias@ics.ufpa.br (A.G.d.S.D.); lucas.gato@ics.ufpa.br (L.d.S.G.); alanna.pimentel@ics.ufpa.br (A.L.P.d.S.); 3 Pharmaceutical Science Post-Graduation Program, Faculty of Pharmacy, Federal University of Pará, Belém 66075-110, Brazil; saviomontsan@gmail.com; 4 Medical School, Medical Science Institute, Federal University of Pará, Belém 66075-110, Brazil; leonardo.alencar@icm.ufpa.br; 5 Laboratory of Cellular and Molecular Immunology, Department of Basic Health Sciences, Federal University of Health Sciences of Porto Alegre, Porto Alegre 90050-170, Brazil; gilsondorneles@gmail.com (G.P.D.); pedror@ufcspa.edu.br (P.R.T.R.); 6 Department of Food Engineering, Midwest State University-UNICENTRO, Simeao de Camargo Varela de Sá, 03, Guarapuava 85.040-080, Brazil; hertastutz@gmail.com (H.S.); vanessasovrani@gmail.com (V.S.)

**Keywords:** mushroom, sepsis, *Agaricus brasiliensis*, cecal ligation and puncture, antioxidant, immunomodulator

## Abstract

Sepsis is a potentially fatal clinical condition that results from an immune imbalance in the host during an infection. It presents systemic alterations due to excessive activation of pro-inflammatory mediators that contribute to inflammation, formation of reactive species, and tissue damage. Anti-inflammatory mediators are then extensively activated to regulate this process, leading to immune exhaustion and, consequently, immunosuppression of the host. Considering the biological activities of the nutraceutical *Agaricus brasiliensis* (*A. brasiliensis*), such as immunomodulatory, antioxidant, and antitumor activities, the present study investigated the therapeutic potential of the lipid fraction of *A. brasiliensis* (LF) in a model of lethal sepsis in mice (*Mus musculus*), induced by cecal ligation and perforation (CLP). The results showed that treatment of septic animals with LF or LF associated with ertapenem (LF-Erta) reduced systemic inflammation, promoting improvement in clinical parameters and increased survival. The data show a reduction in pro-inflammatory and oxidative stress markers, regulation of the anti-inflammatory response and oxidizing agents, and increased bacterial clearance in the peritoneal cavity and liver. Thus, it can be concluded that LF as a treatment, and in conjunction with antibiotic therapy, has shown promising effects as a hepatoprotective, antioxidant, antimicrobial, and immunomodulatory agent.

## 1. Introduction

Sepsis is a potentially fatal organ dysfunction that is secondary to a dysregulated host immune response to a local infection [1,2]. The World Health Organization has shown that it affects more than 200 people per year for every 100,000 people alive, with a mortality of almost 30% in hospitals and more than 40% in intensive care units [3]. The immune response in sepsis begins with a storm of pro-inflammatory cytokines that activate the innate immune system, sustaining an exacerbated inflammatory state [4]. Simultaneously, anti-inflammatory cytokines contribute to a state of immunosuppression [5], which can lead to death in hospitals due to secondary infections and organ damage as a result of a hyper-inflammatory state [6]. Although there has been significant advancement in understanding the pathology of sepsis, the clinical management of sepsis is challenging due to difficulties in diagnosis, a lack of reliable prognostic biomarkers, and treatment options that are largely limited to antibiotic therapy and fundamental supportive care. Thus, there is an urgent need for the discovery of new therapies [7].

In this study, the intestine was the infectious focus of sepsis through cecal ligation and puncture (CLP) in an animal model (*Mus musculus*). The bacteria enter the bloodstream through the high vascularity of the intestine, reaching the liver (which is also highly vascularized and is another focus of this study). This organ has a significant role in metabolism, digestion, detoxification, and elimination of substances from the body [8], fulfilling an important role in the metabolization of drugs. In that regard, liver alterations, which are found in about 46% of septic patients [9], can compromise drug metabolism and affect drug pharmacokinetics and may lead to liver failure [8].

Currently, there is a growing interest in exploring natural products for the treatment of several diseases, as some traditional medicines have been associated with potential adverse effects. These natural antimicrobial compounds are extracted from different sources and have been demonstrated to be effective against a variety of diseases. These compounds have shown promise in reducing the microbial diseases linked to the development of drug tolerance and resistance [10]. In a previous study [11], our group demonstrated that the aqueous extract of *Agaricus brasiliensis* (*A. brasiliensis*), which is a functional food with a high number of bioactive components, proteins, lipids, minerals, and vitamins, was able to increase the survival of septic animals through a mechanism involving immunomodulatory and antioxidant protective effects. This was mainly because of the lipid fraction of edible Basidiomycete fungus such as *A. brasiliensis*, have a high amount of ergosterol is found [12,13].

In our previous study, the whole mycelium of *A. brasiliensis* was tested; however, in this study, only lipid fraction of *A. brasiliensis* (LF) was used, which is rich in ergosterol. According to the previous study [11], the integral mycelium of the mushroom demonstrated a control of local (hepatic) and generalized infections. The current study showed that the therapeutic effects of this isolated fraction were better, being able to reduce the generation of reactive oxygen species (ROS) 6 and 24 h after CLP, inhibit the production of malondialdehyde (MDA) in 24 h, and reverse the decrease in glutathione (GSH) levels in 24 h. LF can be used in monotherapy or as a therapeutic supplement along with standard antibiotics, such as ertapenem (one of the most commonly used in urgent and emergency infections), which was used in this study.

Therefore, considering sepsis as one of the major global public health challenges and knowing the immunomodulatory, antimicrobial, and systemic antioxidant effects of *A. brasiliensis*, this study aimed to demonstrate the efficacy of the ergosterol-rich LF of the mushroom in the improvement of clinical parameters of sepsis, inflammatory status, markers of oxidative stress, bacterial load in the inflammatory site and liver, and survival of the animals.

## 2. Material and Methods

### 2.1. Ethics Statement

This study was carried out in strict accordance with the current national legislation on Procedures for the Scientific Use of Animals (Federal Law No. 11,794, of 8 October 2008) and the Brazilian Guideline for the Care and Use of Animals for Scientific Purposes and Didactics of the National Council for the Control of Animal Experimentation—CONCEA (CONCEA, 2013). All procedures used in this study were approved by the institutional Committee for Animal Ethics of Federal University of Pará/UFPA (n° 8355050418).

### 2.2. Preparation of Lipid Fraction from A. brasiliensis (LF)

LF was prepared and obtained in collaboration with Prof. Dr. Herta Stutz from the Bioprocesses Laboratory, Food Engineering Department, UNICENTRO, Paraná, Brazil. The inoculum was prepared from *A. brasiliensis* mycelium [14] and cultivated on *Ilex paraguariensis*. The extraction process involved grinding the samples to 60 mesh, determining initial moisture content, and treating 250 g of the sample with methanol and NaOH in screw-capped tubes. The mixture was heated in a microwave, cooled, and sequentially treated with ethanol, then centrifuged, and the supernatant removed. Ethanol was evaporated at 45 °C under aeration. Ergosterol quantification was carried out using a spectrophotometer (281 nm) [15]. The ergosterol-rich lipid fraction was lyophilized and provided by Prof. Dr. Stutz. In LABEIM (UFPa—Belém, PA, Brazil), a stock solution (26.5 mg/mL) of lyophilized LF, was prepared in 10% of dimethylsulfoxide (DMSO, Dinâmica Química Contemporânea Ltda., Indaiatuba, SP, Brazil) and sterile distilled water. To determine the correct LF volume for administration to mice at a dose of 0.2 mg·kg^−1^, animals were weighed before dosing. This dose was chosen based on *in vitro* cytotoxicity tests using Raw-Luc 264.7, as well as antioxidant and immunomodulatory activities (unpublished data).

### 2.3. Experimental Design

Male and female Swiss mice (*Mus musculus*) (n = 75) aged 30 to 90 days old and weighing 20 to 30 grams were obtained from the Central Animal Facility of the Institute of Biological Sciences at UFPA. All animals were housed under controlled temperature conditions (25 ± 1 °C) and were maintained on a 12 h light/dark cycle (6–18 h), with *ad libitum* access to food and water. All animals were acclimatized to conditions for 3 days before use.

**Scheme 1:**
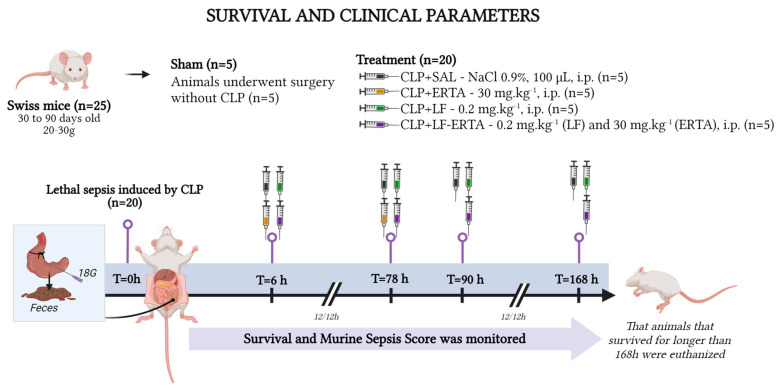
Experimental design of lethal CLP model and treatments. Created with BioRender.com (Agreement number: BL273VUPE5).

In this study, for the evaluation of a survival and murine sepsis score (MSS), 25 animals (Scheme 1) were randomly divided into the following (n = 5/group):

1. Sham group: animals underwent surgery without CLP (n = 5);
2. Saline solution 0.9% (CLP+SAL) group: mice were subjected to lethal sepsis by CLP and treated with saline solution (n = 5);
3. Ertapenem (CLP+ERTA) group: mice were subjected to lethal sepsis by CLP and treated with 30 mg·kg^−1^ of ertapenem (n = 5);
4. LF (CLP+LF) group: mice were subjected to lethal sepsis by CLP and treated with 0.2 mg·kg^−1^ of LF (n = 5);
5. Ertapenem and LF (CLP+LF-ERTA) group: mice were subjected to lethal sepsis by CLP and received 30 mg·kg^−1^ of ertapenem and 0.2 mg·kg^−1^ of LF (n = 5).

All animals that were induced with lethal sepsis were treated 30 min, 6, and 12 h/12 h after CLP for 3 days following the treatment protocol of ertapenem proposed by [16].

**Scheme 2:**
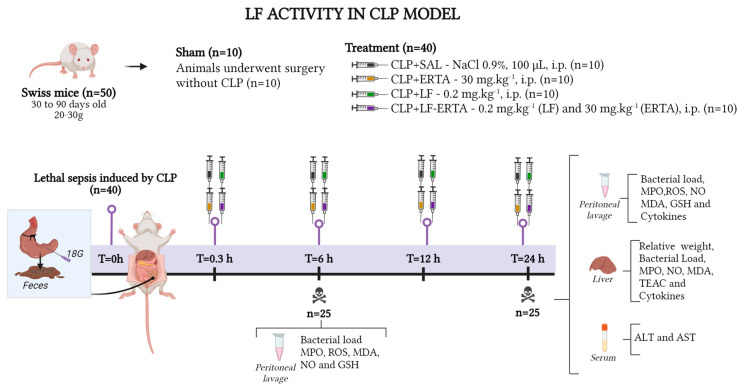
Experimental protocol of evaluation of LFAb activity in CLP model. ALT—alanine aminotransferase; AST—aspartate aminotransferase; ERTA—ertapenem; GSH—glutathione; CLP—lethal sepsis by cecal ligation and puncture; LF—lipid fraction of *A. brasiliensis*; LF-ERTA—lipid fraction of *A. brasiliensis* and ertapenem; MDA—malondialdehyde; MPO—myeloperoxidase; NO—nitric oxide; ROS—reactive species of oxygen; TEAC—total evaluation of Trolox equivalent antioxidant capacity. Created with BioRender.com (Agreement number: JF273VUPMS).

To assess antioxidant and immunomodulatory activity in the peritoneal cavity and liver at 6 and/or 24 h after lethal sepsis induced by CLP, 50 animals (Scheme 2) were randomly divided into 5 groups (n = 10/group):

1. Sham group: animals underwent surgery without CLP (n = 10);
2. CLP+SAL group: mice were subjected to lethal sepsis by CLP and treated with saline solution (n = 10);
3. CLP+ERTA group: mice were subjected to lethal sepsis by CLP and treated with 30 mg·kg^−1^ of ertapenem (n = 10);
4. CLP+LF group: mice were subjected to lethal sepsis by CLP and treated with 0.2 mg·kg of LF (n = 10);
5. CLP+LF-ERTA group: mice were subjected to lethal sepsis by CLP and received 30 mg·kg^−1^ of ertapenem and 0.2 mg·kg^−1^ of LF (n = 10).

A total of 25 animals (n = 5/group) were treated 30 min and 6 h after CLP and 25 animals (n = 5/group) were treated 30 min, 6, and 12 h after CLP, until 24 h when they were submitted to euthanasia. After 6 and 24 h after CLP, the animals were anesthetized under aseptic conditions and submitted to euthanasia by cardiac puncture to collect the samples (peritoneal lavage, liver, and serum). The experimental procedure is summarized in (Scheme 2).

### 2.4. Lethal CLP Model 

The induction of polymicrobial sepsis in mice via cecal ligation and puncture (CLP), as conducted in our prior studies [11,16,17] followed a protocol adapted from [18]. Animals were anesthetized intraperitoneally with a combination of ketamine (100 mg·kg^−1^) and xylazine (10 mg·kg^−1^). Upon confirming anesthesia and the absence of paw reflex, animals were positioned on a surgical table with their head and cervical region extended, and ophthalmic lubricant was applied to prevent corneal desiccation. Following abdominal shaving, the surgical site was aseptically prepared using 70% isopropyl alcohol and 2% betadine. A midline 1 cm incision was made through the skin, fascia, and abdominal muscle to expose the cecum. The cecum was ligated near the ileocecal valve using a 3-inch silk suture, ensuring minimal risk of ischemia. Subsequently, lethal sepsis was induced by a single puncture of the cecum with an 18-gauge needle, followed by extrusion of approximately 1 cm of fecal material from each puncture site.

After carefully returning the cecum to the abdominal cavity, the incision was closed with silk sutures. Sham-operated mice underwent an identical procedure, with the exception that the cecum was not ligated or punctured. Following surgery, all animals received a subcutaneous injection of 1 mL of 0.9% saline to prevent hypovolemia and were maintained under warming conditions; moreover, mice were monitored continuously until able to right themselves as a confirmation of anesthetic recovery.

### 2.5. Survival and Evaluation of Clinical Parameters 

The survival rate was determined in relation to the time elapsed after the procedure. The objective was to evaluate the physiological changes in mice submitted to CLP. To gauge the severity of sepsis during the 168 h (7 days) of the survival assessment, the MSS scoring system was used according to [19] and adapted by [20], which mimics the protocols used in human sepsis, such as the sequential organ failure assessment (SOFA). The assessment of the animals was conducted by three researchers in a single-blind manner, where only those handling the cages were aware of the group identity. Initially, evaluations were conducted every hour after CLP time, followed by assessments every 2 h for up to 24 h after CLP. Subsequently, the animals were evaluated twice a day until the completion of the seven-day survival assessment. Each animal was individually evaluated for the following variables: appearance (degree of piloerection), eyes (closed or presence of secretions), level of consciousness (active or non-responsive), spontaneous activity, response to tactile and auditory stimuli, and quality of breathing. They were classified with points between 0 and 3 and, at the end of each evaluation, the MSS was calculated based on the sum of the score in different time points for each animal in the same group. The mice were euthanized if the respiratory quality score was 3 at any time. In addition, the animals were monitored twice a day for parameters such as body weight and temperature and water and food intake.

### 2.6. Blood Samples and Peritoneal and Liver Collection

After sedation, approximately 1 mL of blood was collected by cardiac puncture 6 or 24 h after CLP induction, as described by [21]. The blood sample collected was distributed into microtubes to assess biochemical parameters (for example, cytokine levels). To obtain serum, the blood was centrifuged at 3000 rotations per minute (RPM) for 15 min. The resulting serum sample was separated and stored at −80 °C until analysis.

After blood collection, the animals underwent peritoneal lavage with sterile 1× phosphate buffered saline (PBS). One part of the sample from this lavage was separated for bacterial load analysis and the other was placed in 1.5 mL microtubes and centrifuged at 3000 RPM for 10 min, after which the supernatant was collected to assess oxidative stress parameters.

Right after, the animals’ livers were collected, weighed, and washed with PBS 1×. Subsequently, the livers were macerated with PBS 1×, and this mixture was centrifuged for 10 min at 3000 RPM, allowing the supernatant to be collected in microtubes and separated into two parts. The first part of the organ was diluted 1:10 for the determination of colony-forming units (CFU), and the second part was stored in a −80 °C freezer until analysis.

### 2.7. Bacterial Load Determination 

For the determination of colony-forming units (CFU) in the liver and peritoneal fluid of mice from the Sham and treatment groups, 10 μL of the sample was diluted with sterile PBS at a ratio of 1:10. Subsequently, 10 μL of each dilution was cultured on Müller–Hinton agar (HIMEDIA, Londrina, PR, Brazil) and incubated at 37 °C for 24 h. The colonies were then counted and expressed as CFU/mL.

### 2.8. Myeloperoxidase (MPO) Dosage

The samples were collected 6 or 24 h after CLP induction, washed with 11× PBS, sonicated with a buffer solution, and then centrifuged. Subsequently, the obtained supernatant was utilized to measure the MPO produced by polymorphonuclear leukocytes [22]. MPO was assessed through a peroxidase reaction with the chromogenic substrate 3, 3′, 5, 5′-tetramethylbenzidine (TMB, Sigma Chemical Co., St. Louis, MO, USA), following the procedure outlined by [23] and adapted by [22]. In a well, 70 µL of TMB solution (2.9 mM in dimethylsulfoxide; 14.5% DMSO), 50 µL of 0.75 mM H_2_O_2_, 10 µL of sodium phosphate buffer (150 mM at pH 5.4), and 20 µL of the sample were incubated in a water bath at 37 °C for 10 min. The reaction was terminated by adding 10 µL of 2 M sulfuric acid, and the absorbance was read in the Synergy HTX Multimode Reader (BioTek, Winooski, VT, USA) at 450 nm. 

### 2.9. Quantification of Intracellular Reactive Oxygen Species (ROS) Release

The peritoneal lavage was centrifuged at 1800× *g* for 10 min and then cells were washed twice in PBS. After washing, 10 µM dichlorodihydrofluorescein 2,7-diacetate (H_2_DCFH-DA, Sigma Chemical Co., St. Louis, MO, USA), a non-fluorescent compound that passively diffuses through biological membranes, was added. H_2_DCFH-DA is hydrolyzed by intracellular esterases to dichlorodihydrofluorescein (DCF), a compound that generates fluorescent by-products after interacting with ROS present in the intracellular environment. Thus, the ROS content was measured by DCF oxidation in a spectrofluorometer at emission and excitation wavelengths of 525 nm and 450 nm, respectively.

### 2.10. Determination of Nitric Oxide (NO) Production 

NO was measured through the concentration of nitrite in the samples, using the Griess reaction, in which diazotization occurs in an acidic medium (pH 2.5). The diazo compound formed reacts with N-(1-naphthyl) ethylenediamine hydrochloride (NED), producing a reddish-pink compound. At room temperature, 100 μL of the sample was incubated with 100 μL of Griess reagent and, after 10 min, the reaction product was measured under absorbance at 550 nm. The NO_2_ concentration was determined using a standard curve and expressed in μM [24].

### 2.11. Malondialdehyde (MDA)

MDA was quantified using the thiobarbituric acid reactive substances (TBARS) method [25] adapted by [26], the principle of which lies in the chemical bond between the MDA and TBA molecules, which results in the formation of a slightly pink chromophore. The samples were previously treated with 0.05 M trichloroacetic acid (TCA, Sigma-Aldrich, St. Louis, MO, USA) to precipitate lipoproteins and then with TBA (10 nM). The complexation of lipid peroxide with TBA was facilitated by heating in a water bath at 94 °C for 60 min. The resulting chromogen was extracted in n-butanol and measured by absorbance at a wavelength of 535 nm. The MDA standard (1, 1, 3, 3, tetrahydroxypropane, Sigma-Aldrich, St. Louis, MO, USA) was used to make the standard curve, and the results were expressed in nmol/L

### 2.12. Glutathione (GSH) Levels 

The GSH level was determined in peritoneal lavage fluid samples from septic mice 6 and 24 h after CLP induction using Ellman’s reagent, following an adapted method from [27]. This test is based on the production of yellow color when 5, 5′-dithiobis (2-nitrobenzoic acid) (DTNB, Sigma-Aldrich, St. Louis, MO, USA) is added to compounds containing sulfhydryl groups. The GSH concentration was determined using a standard curve constructed with different concentrations of GSH in the reduced form. The absorbance was recorded at 412 nm on a microplate reader (SpectraMax 250, Molecular Devices, Union City, CA, USA), and the results were expressed in μmol/mL.

### 2.13. Determination of Aspartate Aminotransferase/Serum Glutamic Oxaloacetic Transaminase (AST/SGOT) and Alanine Aminotransferase/Serum Glutamic Pyruvic Transaminase (ALT/SGPT)

To assess liver function, AST/SGOT and ALT/SGPT enzymes were measured in mouse serum 24 h after CLP, obtained by centrifuging 200 μL of blood at 4750× *g* for 10 min. Subsequently, the activity of the liver enzymes was measured by the kinetic method, AST/SGOT and ALT/SGPT Liquiform (Labtest ^®^, Lagoa Santa, MG, Brazil), according to the manufacturer’s recommendations.

### 2.14. Cytokine Measurement 

Interleukins IL-1β, IL-17, and IL-10, tumor necrosis factor-alpha (TNF-α), and transforming growth factor-beta (TGF-β) were collected from the samples 24 h after lethal CLP using the commercial kit (R&D Systems, Minneapolis, NE, Canada) according to the manufacturer’s instructions. The results were expressed in pg/mL.

### 2.15. Total Evaluation of Trolox Equivalent Antioxidant Capacity (TEAC) 

The total antioxidant capacity (TAC) of serum samples, peritoneal fluid, and livers from septic mice (collected 6 and/or 24 h after CLP induction) was assessed using the TEAC assay with [±]-6-hydroxy-2, 5, 7, 8-tetramethylchroman-2-carboxylic acid (Trolox, Sigma-Aldrich, St. Louis, MO, USA). This assay provides relevant information that effectively describes the dynamic balance between pro-oxidant and antioxidant compounds. In this assay, the diammonium salt, 2, 2′-azino-bis(3-ethylbenzothiazoline-6-sulfonic acid) (ABTS, Sigma-Aldrich, St. Louis, MO, USA) was incubated with potassium persulfate (Sigma-Aldrich, St. Louis, MO, USA) to produce ABTS+, a green-blue chromophore. The inhibition of ABTS+ formation by antioxidants in the samples was expressed as Trolox equivalents, determined at 740 nm using a calibration curve plotted with different amounts of Trolox [28,29].

### 2.16. Statistical Analysis

The data were subjected to statistical analysis, using the GraphPad Prism 8 software, where each parameter was initially analyzed for possible outliers using the calculation of the interquartile range. Student’s *t*-test was used to determine the existence of significant differences for each parameter analyzed in each group from the beginning to the end of the study. For each parameter analyzed, a two-way analysis of variance (ANOVA) was performed, followed by the Tukey test for pairwise mean comparisons. Pearson’s correlation test was conducted to assess possible correlations between parameters. Results were considered statistically significant for *p* ≤ 0.05.

## 3. Results

### 3.1. LF Treatment Increases Survival and Improves the Clinical Parameters of SEPTIC Animals

**Figure 1:**
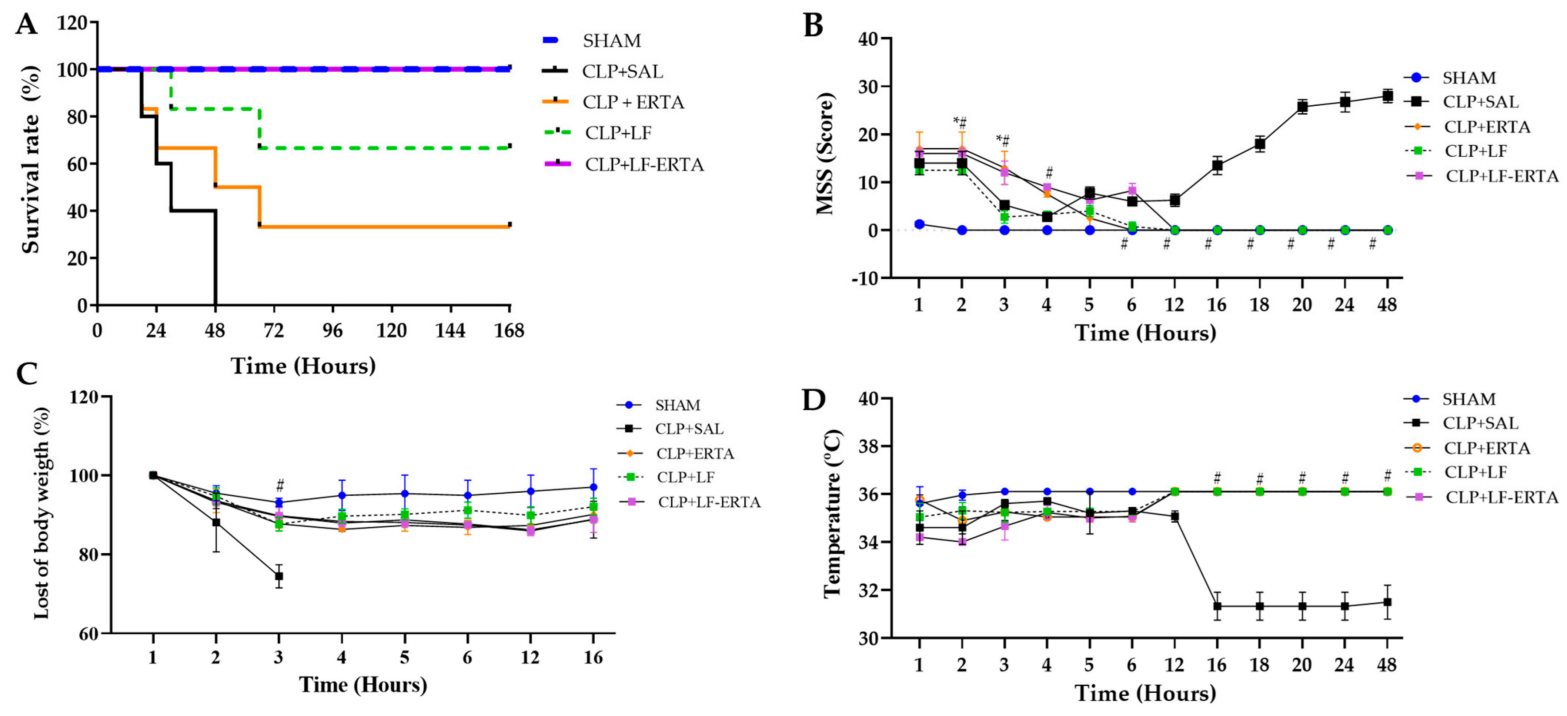
Effect of LF as an isolated or adjuvant therapy on the survival rate (%) and the physiological parameters of septic animals. Survival of animals submitted to CLP and treated with saline (0.9%), ERTA (30 mg·kg^−1/^animal), LF (0.2 mg·kg^−1^/animal), or LF-ERTA (LF: 0.2 mg·kg^−1/^animal; ERTA: 30 mg·kg^−1^/animal). (**A**) Survival rate (%). (**B**) Total sepsis score up to 48 h after CLP. (**C**) Loss of body weight (%). (**D**) Temperature. The survival test was expressed in percentage and evaluated by the log-rank Mantel–Cox test. The evaluation of the clinical parameters was represented by mean ± SD of n = 5 animals. * *p* < 0.05 treatment (ertapenem, LF, or LF with ertapenem) versus Sham group. # *p* < 0.05 treatment (LF or LF with ertapenem) versus CLP-saline group (two-way ANOVA followed by post-Bonferroni test). CLP—cecal ligation and puncture; ERTA—ertapenem; LF—lipid fraction from *Agaricus brasiliensis*; LF-ERTA—lipid fraction from *Agaricus brasiliensis* associated with ertapenem; MSS—murine sepsis score; SAL—saline 0.9%.

As shown in Figure 1, all animals from the CLP+SAL group died within 48 h after sepsis induction (Figure 1A) and, during this period, these animals exhibited worsening clinical parameters with high severity scores (Figure 1B). There was a reduction in water intake (6.0 ± 2.12 mL/day) and food intake (2.0 ± 1.41 g/day), resulting in weight loss (Figure 1C), thus confirming the severity of the model used in this study. Additionally, between 6 and 10 h, these animals displayed reduced movement, squinting, and piloerection, which may indicate pain. Furthermore, they were unresponsive, even when provoked with auditory stimuli, and did not respond to touch. Their eyes had secretions and remained closed most of the time. They also experienced difficulties in breathing and exhibited hypothermia compared with the Sham group, along with elevated clinical scores.

Regarding the CLP+ERTA group, the animals had a survival rate of 33.3% (Figure 1A) and showed improvement in physiological changes until 48 h after CLP induction (Figure 1B). They also exhibited an increase in water intake (8.5 ± 0.70 mL/day) and feed intake (1.5 ± 0.70 g/day) 24 h after CLP induction. On the other hand, the CLP+LF group showed a survival rate of 66.7% (Figure 1A) and significant improvement in physiological changes (Figure 1B). Figure 1C shows a significant reduction in the MSS score 8 h after CLP induction, indicating that the animals were active with a normal appearance and had normal breathing and normal body temperature (Figure 1D). Additionally, water consumption (35.5 ± 0.70 mL/day) and feed consumption (4.5 ± 2.12 g/day) increased significantly within 24 h compared with the CLP+SAL group, indicating an excellent prognosis for these animals. The administration of LF concomitantly with ertapenem (CLP+LF-ERTA group) resulted in a survival rate of 100% (Figure 1A), with improvements in physiological parameters (Figure 1B–D). Water intake increased to 18 ± 8.48 mL/day, and feed intake increased to 15.5 ± 4.94 g/day 24 h after CLP induction.

### 3.2. LF Alone or with Ertapenem Reduces Bacterial Load and Oxidative Stress Parameters in the Peritoneal Cavity

**Figure 2:**
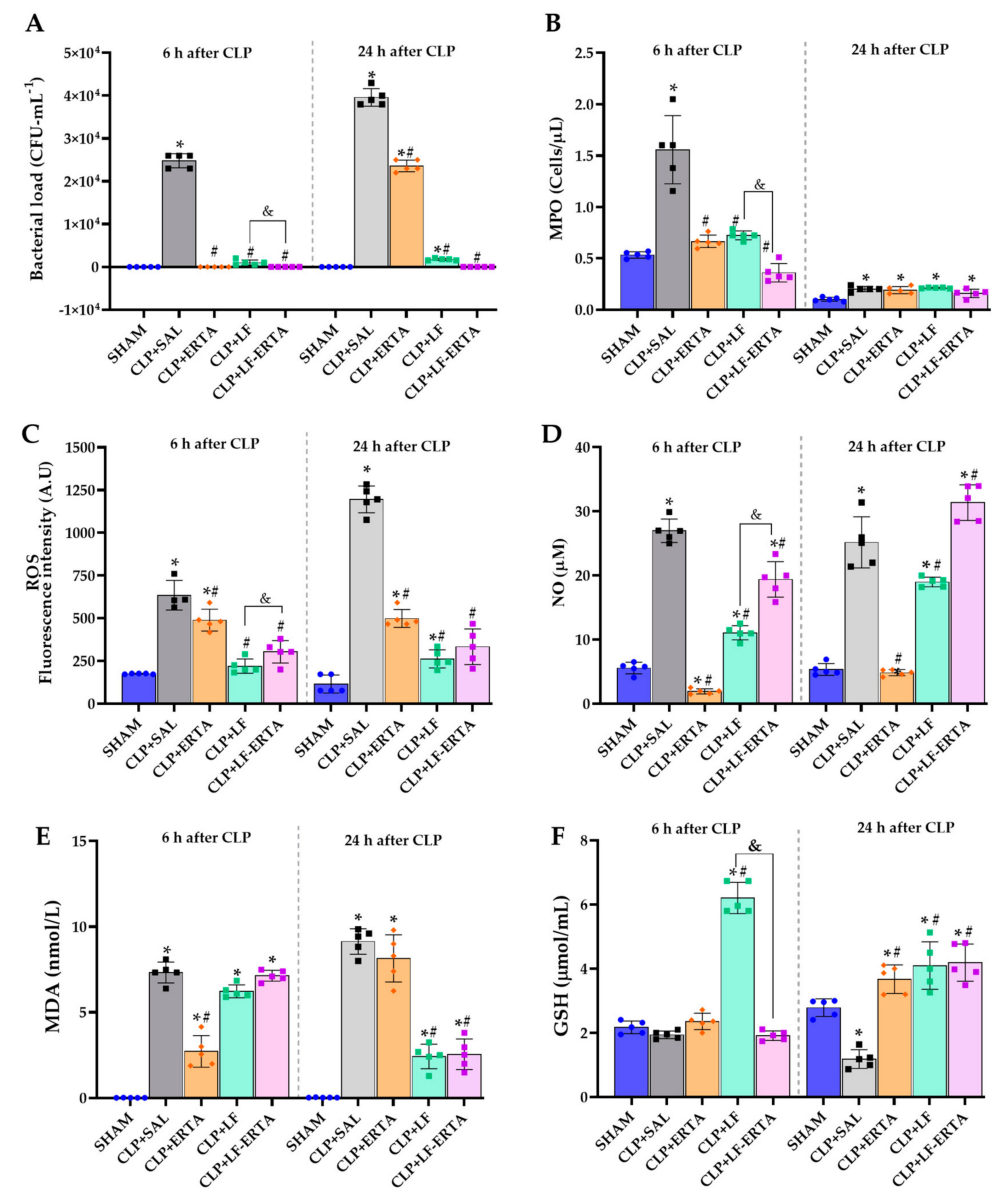
Effect of LF in the peritoneal cavity in a model of lethal sepsis. (**A**) Bacterial load in the peritoneal cavity 6 and 24 h after CLP induction. (**B**) MPO levels 6 and 24 h after CLP. (**C**) ROS generation 6 and 24 h after CLP. (**D**) Nitric oxide levels 6 and 24 h after CLP. (**E**) MDA levels 6 and 24 h after CLP. (**F**) GSH levels 6 and 24 h after CLP in the peritoneal fluid. Data are expressed as mean ± SD (n = 5 per group). * *p* < 0.05 statistically significant differences between treatment (ertapenem, LF, or LF with ertapenem) versus Sham group. # *p* < 0.05 treatment (LF or LF with ertapenem) versus CLP+SAL group. & *p* < 0.05 statistically significant differences between LF versus LF-ERTA (two-way ANOVA followed by post-Bonferroni test). CFU—colony forming unit; CLP—cecal ligation and puncture; GSH—glutathione; ERTA—ertapenem; LF—lipid fraction from *Agaricus brasiliensis*; LF-ERTA—lipid fraction from *Agaricus brasiliensis* associated with ertapenem; MDA—malondialdehyde; MPO—myeloperoxidase; NO—nitric oxide; ROS—reactive oxygen species; SAL—saline 0.9%.

The CLP+SAL group showed a high bacterial load at 6 (24.8 ± 1.64 CFU-mL^−1^) and 24 h (39,600.0 ± 2073.64 CFU-mL^−1^) after CLP. CLP+ERTA reduced the bacterial load in the first 6 h (1.4 ± 1.67 CFU-mL^−1^), but the bacterial load increased 24 h after CLP (23.60 ± 1.34 CFU-mL^−1^). The treatment with LF, either alone or with ertapenem, also reduced the bacterial load at 6 h (CLP+LF = 1000.0 ± 612.37 CFU-mL^−1^; CLP+LF-ERTA = 1.6 ± 1.5 CFU-mL^−1^), but the bacterial load increased at 24 h (CLP+LF = 5660.0 ± 219.08 CFU-mL^−1^; CLP+LF-ERTA = 1748.0 ± 241.5 CFU-mL^−1^); however, LF-ERTA showed better antimicrobial activity than LF (Figure 2A). The increase in bacterial load in the peritoneal cavity resulted in the recruitment of leukocytes. In this regard, the CLP+SAL group showed an increase in the recruitment of leukocytes in the peritoneal cavity and an increase in the MPO enzyme at 6 h (2.0 ± 0.32 cells/μL). On the other hand, the CLP+ERTA (6 h = 0.66 ± 0.08 cells/μL; 24 h = 0.159 ± 0.002 cells/μL), CLP+LF (6 h = 0.72 ± 0.08 cells/μL; 24 h = 0.16 ± 0.006 cells/μL), and CLP+LF-ERTA (6 h = 0.37 ± 0.123 cells/μL; 24 h = 0.2 ± 0.025 cells/μL) groups had reduced MPO enzyme levels compared with the CLP+SAL group 6 h after CLP (Figure 2B)

The inflammatory process of sepsis increased the generation of ROS (Figure 2C) and NO (Figure 2D) in the CLP+SAL group 6 h after CLP; however, LF (isolated or in combination with ertapenem) reduced ROS generation at both time points (Figure 2C). Only the treatment with ertapenem and LF (isolated) reduced NO levels 6 and 24 h after CLP. In contrast, CLP+LF-ERTA increased NO levels 24 h after sepsis induction (Figure 2D); however, the treatments protected these animals. Ertapenem inhibited the production of MDA at 6 h, while LF and LF-ERTA inhibited MDA production at 24 h (Figure 2E). As a consequence of the oxidative cellular environment at the inflammatory site, animals with sepsis showed a decrease in endogenous antioxidants such as GSH at 24 h (Figure 2F). The treatments with LF and/or ERTA were able to reverse the decrease in GSH, especially at 24 h (Figure 2F).

### 3.3. LF Alone or with Ertapenem Exhibits Anti-Inflammatory Action in the Peritoneal Cavity

**Figure 3:**
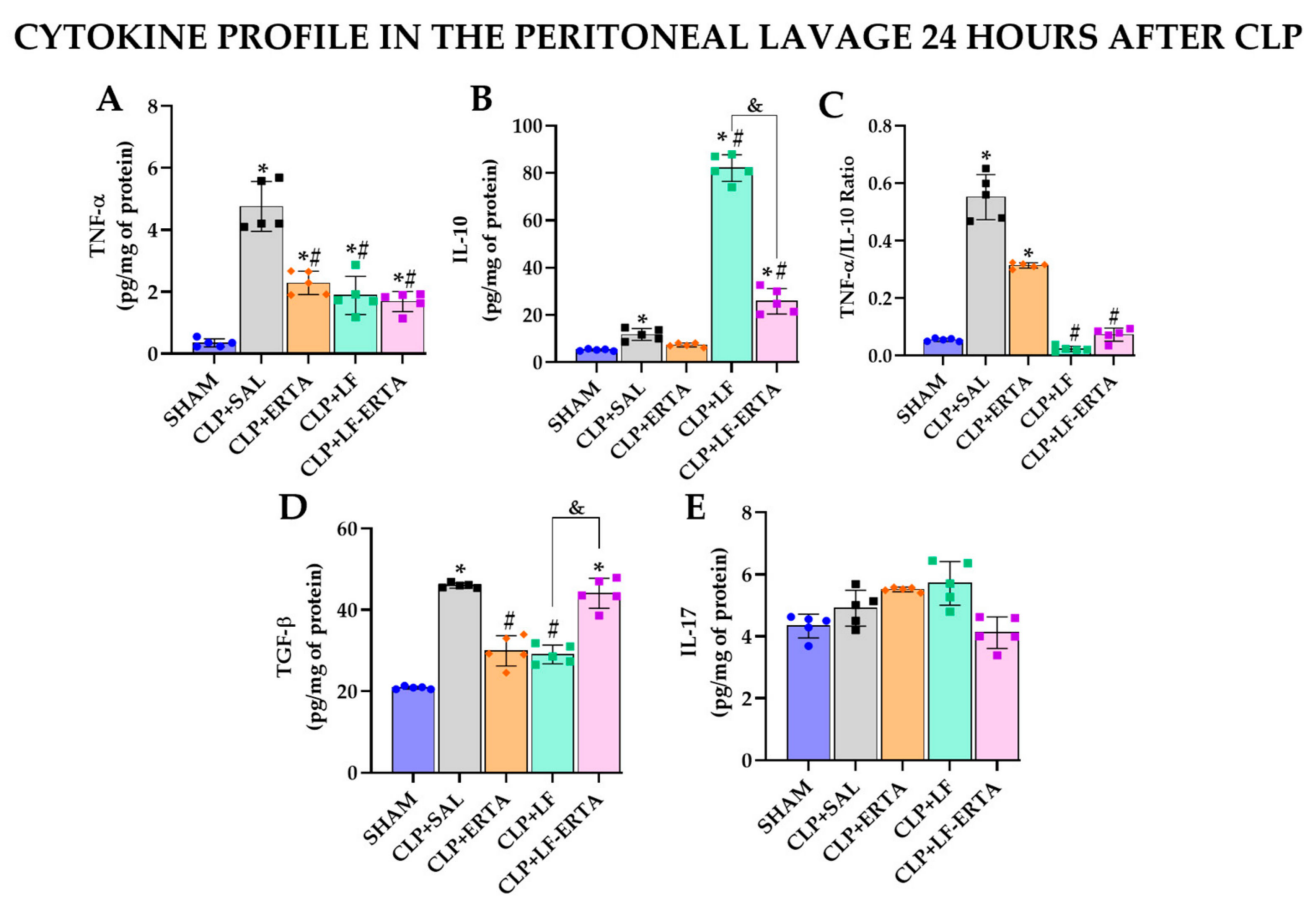
The effect of treatment with LF alone or concomitantly with ertapenem on the cytokine profile in the peritoneal cavity. (**A**) Levels of TNF-α, (**B**) IL-10, (**C**) TNF-α/IL-10 ratio, (**D**) TGF- β, and (**E**) IL-17 in the peritoneal cavity 24 h after CLP. Data are expressed as mean ± SD (n = 5 per group). * *p* < 0.05, statistically significant differences between treatment (ERTA, LF, or LF-ERTA) versus Sham group. # *p* < 0.05 treatment (LF or LF-ERTA) versus CLP+SAL group. & *p* < 0.05 treatment LF vs. LF-ERTA. CLP—cecal ligation and puncture; ERTA—ertapenem; LF—lipid fraction from *Agaricus* brasiliensis; LF-ERTA—lipid fraction from *Agaricus brasiliensis* associated with ertapenem; SAL—saline 0.9%.

Regarding the cytokine profile at 24 h, the CLP+SAL group exhibited an inflammatory profile with increased TNF-α and TGF-β, while the treatments reduced these cytokines (Figure 3A,D). In terms of IL-10, treatment with LF alone or with ERTA elevated the levels of this cytokine (Figure 3B). The TNF-α/IL-10 ratio showed that the CLP+SAL group was elevated (0.56 ± 0.1 pg/mL) compared with the CLP+ERTA group (0.31 ± 0.01 pg/mL) and the treated groups, CLP+LF (0.02 ± 0.01 pg/mL) and CLP+LF-ERTA (0.07 ± 0.03 pg/mL), with no statistically significant difference compared with the Sham group (0.05 ± 0.006 pg/mL) (Figure 3C). The levels of IL-17 in the peritoneal cavity did not show any significant changes in the treatment groups (ERTA, LF, and LF-ERTA) when compared with the Sham and CLP+SAL groups (Figure 3E).

### 3.4. LF Alone or with Ertapenem Have Antimicrobial and Antioxidant Activity in the Liver 

**Figure 4:**
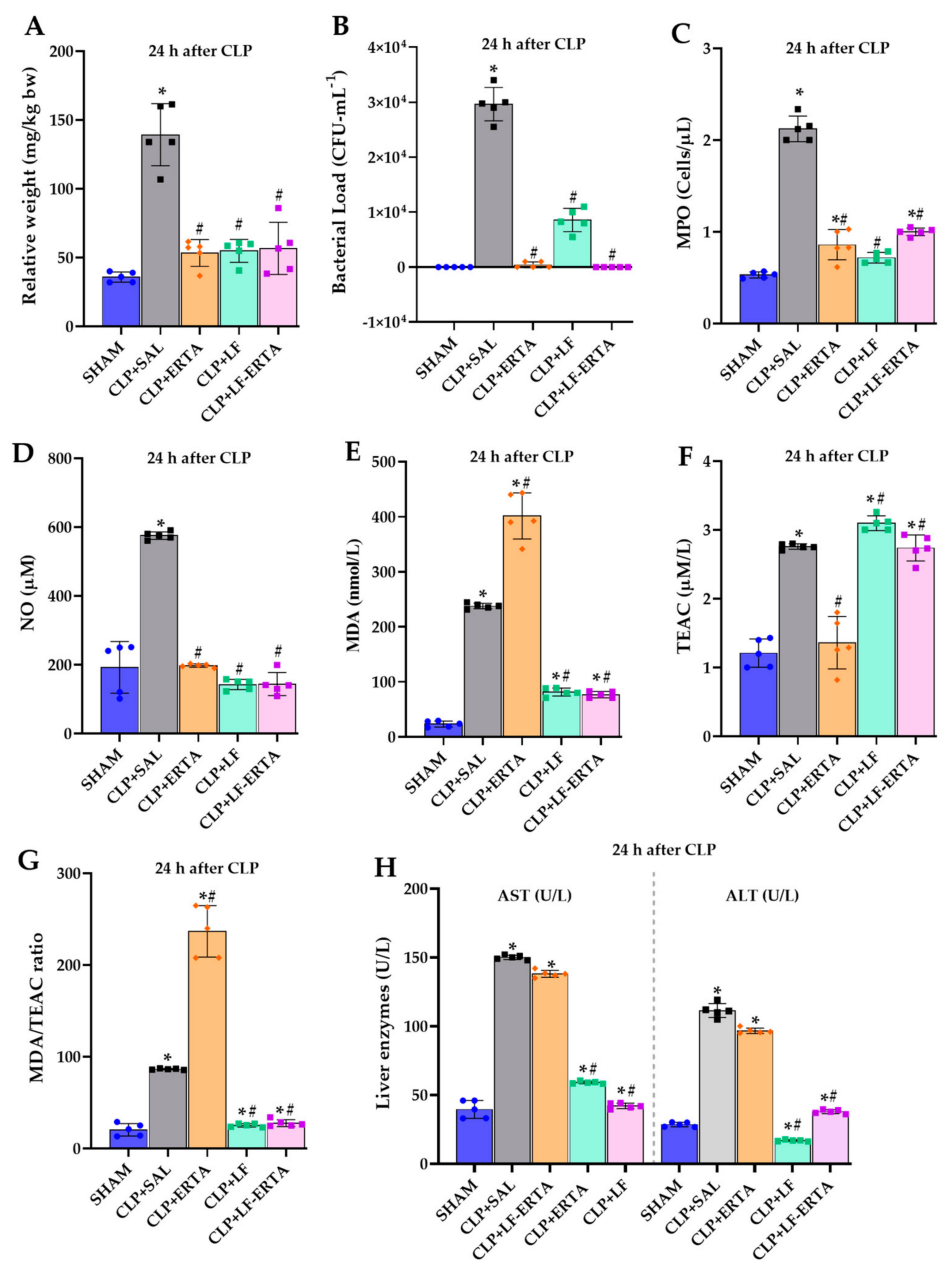
Effect of FLAb on sepsis-induced hepatic dysfunction. (**A**) Relative weight. (**B**) Bacterial load in the liver. (**C**) MPO levels in the liver. (**D**) Nitric oxide levels in the liver. (**E**) MDA levels in the liver. (**F**) TEAC levels in the liver. (**G**) MDA/TEAC ratio. (**H**) AST and ALT. * *p* < 0.05 treatment (FLAb or FLAb with ertapenem) versus CLP group. Data are expressed as mean ± SD (n = 5 per group). * *p* < 0.05, statistically significant differences between treatment (ERTA, LF, or LF-ERTA) versus Sham group. # *p* < 0.05 treatment (LF or LF-ERTA) versus CLP+SAL group. CLP—cecal ligation and puncture; ERTA—ertapenem; LF—lipid fraction from *Agaricus brasiliensis*; SAL—saline 0.9%.

Twenty-four hours after CLP, the CLP+SAL group exhibited the highest relative liver weight (134.1 ± 27.32 mg·kg^−1^) compared with other groups treated with LF (CLP+LF = 59.93 ± 9.60 mg·kg^−1^; CLP+ERTA = 56.61 ± 21.87 mg·kg^−1^) (Figure 4A). Additionally, the CLP+SAL group showed a high bacterial load (29,750.0 ± 6010.0 CFU-mL^−1^), whereas simultaneous treatment with LF and ERTA reduced the bacterial load 24 h after sepsis induction (0.5 ± 0.7 CFU-mL^−1^). This was a better outcome than when LF was administered alone (8250.0 ± 3889.0 CFU-mL^−1^) (Figure 4B). An increase in bacterial load was associated with an elevation in MPO (Figure 4C) in the CLP+SAL group (2.229 ± 0.04 cells/µL), whereas the CLP+ERTA (0.825 ± 0.11 cells/µL), CLP+LF (0.72 ± 0.08 cells/µL), and CLP + FERTA (0.995 ± 0.03 cells/µL) groups showed a reduction in MPO in the liver.

In relation to oxidative stress parameters, the CLP+SAL group exhibited elevated levels of NO (575.6 ± 14.85 µM) and MDA (237.8 ± 6.6 nmol/L). Although the septic group demonstrated high TEAC levels (2.75 ± 0.07 μM/L), an oxidative imbalance can be observed in the MDA/TEAC ratio, as depicted in Figure 4G. Conversely, septic animals treated with CLP+ERTA, CLP+LF, and CLP+LF-ERTA experienced a reduction in NO levels (199.3 ± 3.7 µM, 143.8 ± 10.61 µM, and 144.6 ± 19.35 µM, respectively) (Figure 4D); however, only the CLP+LF (79.93 ± 8.9 nmol/L) and CLP+LF-ERTA groups (76.92 ± 6.0 nmol/L) demonstrated a reduction in tissue damage (Figure 4E). Additionally, treatment with LF (3.13 ± 0.13 µM/L) and LF-ERTA (2.73 ± 0.1 µM/L) increased the antioxidant capacity in septic animals (Figure 4F), resulting in lower MDA/TEAC ratio values (CLP+LF = 24.46 ± 1.8; CLP+LF-ERTA = 28.27 ± 2.0) compared with the CLP+SAL group (86.47 ± 0.82) (Figure 4G), providing protection against tissue damage (Figure 4E,H). The protective activity of treatment with LF could also be observed by the significant reduction in evaluated transaminases 24 h after the induction of sepsis (Figure 4H).

### 3.5. LF Alone or with Ertapenem Exhibits Anti-Inflammatory Action in the Liver

**Figure 5:**
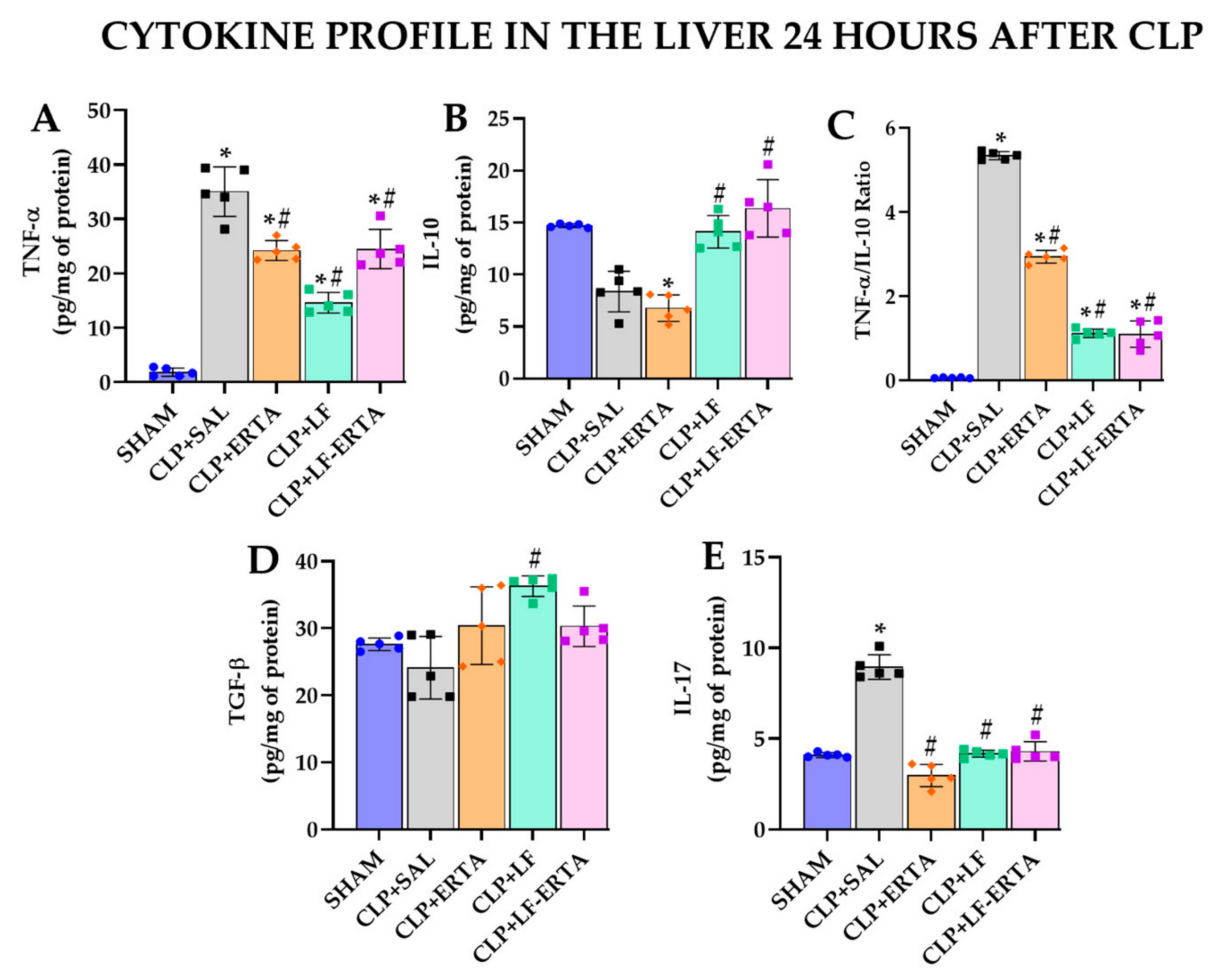
Effect of treatment with LF alone or concomitantly with ertapenem on the cytokine profile in the liver. (**A**) TNF-α, (**B**) IL-10, (**C**) TNF-α/IL-10 ratio, (**D**) TGF-β, and (**E**) IL-17 levels 24 h after CLP. Data are expressed as mean ± SD (n = 5 per group). * *p* < 0.05, statistically significant differences between treatment (ERTA, LF, or LF-ERTA) versus Sham group. # *p* < 0.05 treatment (ERTA, LF, or LF-ERTA) versus CLP+SAL group. CLP—cecal ligation and puncture; ERTA—ertapenem; LF—lipid fraction from *Agaricus brasiliensis*; SAL—saline 0.9%.

In the liver, the CLP+SAL group exhibited an increase in TNF-α 24 h after CLP induction. On the other hand, CLP+ERTA, CLP+LF, and CLP+LF-ERTA showed a reduction in the levels of pro-inflammatory cytokine TNF-α. Notably, the LF group (15.36 ± 1.26 pg/mL) demonstrated the most significant reduction when compared with the CLP+SAL group (34.06 ± 3.26 pg/mL) (Figure 5A). Similarly, the groups treated with LF (14.12 ± 0.9 pg/mL) and LF-ERTA (16.97 ± 1.97 pg/mL) showed increased levels of the anti-inflammatory cytokine IL-10 compared with the untreated CLP+SAL group (8.37 ± 1.11 pg/mL) (Figure 5B). Regarding the TNF-α/IL-10 cytokine ratio, all treated groups (CLP+ERTA, CLP+LF, and CLP+LF-ERTA) exhibited reduced values; however, the CLP+LF (1.12 ± 0.08 pg/mL) and CLP+LF-ERTA (1.07 ± 0.2 pg/mL) groups showed the most favorable values when compared with the CLP+SAL group (5.35 ± 0.11 pg/mL) (Figure 5C). With regard to the levels of cytokine TGF-β, only the CLP+LF group (36.1 ± 1.2 pg/mL) showed a significant increase compared with the CLP+SAL group (22.89 ± 3.09 pg/mL) (Figure 5D). As expected, the CLP+SAL group (9.03 ± 0.53 pg/mL) showed high levels of IL-17 when compared with the Sham group (4.12 ± 0.09 pg/mL), while the treatments (ERTA, LF, and LF-ERTA) favored a reduction in this pro-inflammatory cytokine in the liver 24 h after CLP induction (Figure 5E).

## 4. Discussion

Our study showed evidence of the therapeutic potential of LF of *A. brasiliensis* in a lethal sepsis model in mice. The results revealed that septic animals treated with LF and/or ertapenem exhibited an increase in survival and had an improved prognosis compared with animals that received only ertapenem. The inflammatory site (peritoneum) showed an inhibition in inflammatory status and oxidative stress markers and a decrease in bacterial load. Treatment with LF and/or ERTA also showed a hepatoprotective effect 24 h after sepsis induction. Thus, it can be concluded that LF has a potential immunoprotective and antioxidant effect, contributing to the survival of the animals.

Our previous studies showed that natural products such as the aqueous extract of *A. brasiliensis,* β-Lapachone, or salivary gland extract from *Aedes aegypti,* which has immunomodulatory and antioxidant properties, also increased the survival of septic animals [11,16,17]. In this regard, LF was obtained from the mycelium of *A. brasiliensis* cultivated on *Ilex paraguariensis*, and its effect was evaluated in this study.

In experimental models, the prognosis index is assessed through MSS (murine sepsis score) or M-CASS (mouse clinical assessment score for sepsis) [19]. In the present study, animals subjected to lethal CLP presented a worsening clinical course characterized by piloerection, no response to touch stimuli, and eyes that were mostly closed with the increase in MSS. On the other hand, animals subjected to lethal CLP and treated with LF showed a reduction in MSS. Three hours after CLP, the animals did not exhibit piloerection, potentially indicating reduced pain, and they were active with slightly suppressed activity, had open eyes, and slightly reduced breathing. In this sense, the results found in this study align with the outcomes of a standardized MSS protocol in septic animals, demonstrating a moderate negative correlation between MSS/body temperature and weight loss in the CLP+SAL groups; however, in animals that received treatment, this correlation was divergent, meaning that there was no negative correlation of clinical parameters in these groups. These findings reinforce the therapeutic properties of LF with increased survival [30].

In the early stages of sepsis, the increased bacterial load and its products in the peritoneal cavity activate leukocytes, such as monocytes, and enhance the recruitment of neutrophils to the inflammatory site. The leukocyte activation induces an excessive production of inflammatory mediators and ROS, establishing a pro-oxidant state. In this condition, the organism exhibits an imbalance between the formation and neutralization of free radicals, resulting in the saturation of endogenous antioxidant agents such as GSH [31]. Elevated levels of free radicals and decreased antioxidant agents increase the occurrence of oxidative damage to macromolecules and cellular structures, potentially leading to cell death, as observed previously in our study using a murine sepsis model [17]. Additionally, neutrophils contribute to pathogen elimination through the release of enzymatic granules, including MPO. MPO is an enzyme expressed in leukocytes that stimulates the production of ROS, which plays an important role in eliminating pathogenic bacteria during sepsis; however, studies show that elevated levels of MPO are associated with organ dysfunction due to increased oxidative stress associated with metabolic abnormalities such as the generation of lipoproteins. The effects caused by an excess of MPO lead endothelial cells to express tissue factor, amplifying the risk of thrombosis and systemic inflammatory processes [32].

In this study, the intraperitoneal administration of LF demonstrated antimicrobial activity that did not appear to be dependent on the release of MPO by neutrophils in the peritoneal cavity. Previously, [33] showed that the extract of *Agaricus*, rich in ergosterol, exhibited excellent bacteriostatic activity against both Gram-negative and Gram-positive bacteria found in the gastrointestinal tract of mice and humans, including species such as *Escherichia coli*, *Staphylococcus aureus*, and *Salmonella enteritidis*. The literature also suggests an additional hypothesis, in which ergosterol has the ability to enhance the antimicrobial effect of antibiotics, such as aminoglycosides, contributing to a potential additive effect and making the therapy more effective. It is believed that ergosterol may alter the bacterial membrane, facilitating and enhancing antibiotic uptake [34].

Indeed, LF decreased levels of NO, ROS, and MDA, while increasing the antioxidant GSH 24 h after CLP induction, thereby protecting the animals from oxidative damage. Recently, [35] proposed four mechanisms of the antioxidant activity of sterols in preventing lipid peroxidation: (i) hydrogen atom transfer from the sterol to the free radical, (ii) electron transfer from the sterol to the free radical, (iii) electron transfer followed by proton transfer to the free radical, and, finally, (iv) the formation of a radical adduct, which involves the binding of a radical to a sterol, providing stability to the radical.

Another antioxidative mechanism discussed in the literature involves sterols increasing the expression of nuclear factor erythroid 2 (Nrf-2). Furthermore, Nrf-2 enhances the expression of the enzyme hemeoxygenase-1 (HO-1), which plays a crucial role in inflammation by protecting against oxidative stress and cellular damage [36]. Indeed, the immunomodulatory activity of LF was observed 24 h after sepsis induction at the inflammatory site, as evidenced by the reduction in TNF-α and TGF-β levels. Furthermore, the TNF-α/IL-10 ratio decreased in both groups (CLP+LF and CLP+LF-ERTA), suggesting a potential reduction in the inflammatory process associated with sepsis. These results indicate that the reduction in pro-inflammatory cytokines such as TNF-α may be associated with the fact that ergosterol inhibits the NF-κB/p65 signaling pathway by increasing the expression of Nrf-2. Thus, Nrf-2 negatively regulates the NF-κB signaling pathway by reducing intracellular ROS levels and inhibiting the degradation of IκB-α, and inhibiting the nuclear translocation of NF-κB [37,38].

Regarding the reduction in TGF-β and the increase in IL-10, this may characterize a favorable prognosis for septic animals treated with LF. This is because the increase in TGF-β in late sepsis is associated with higher mortality, reduced bacterial clearance, and increased tissue injury. On the other hand, the increase in IL-10 promotes the expansion of Treg cells and inhibits the production of pro-inflammatory cytokines [39]. As observed by [40], patients who survive sepsis show an increase in IL-33, inducing the differentiation of M2-type macrophages, which secrete IL-10. As a result of tissue damage in sepsis, IL-33 is released, promoting an immunosuppressive response due to the excessive stimulation of anti-inflammatory mediators [41]. The same pattern is observed in lethal sepsis models in mice. However, treatment with LF modulated the inflammatory microenvironment, helping to reduce animal mortality.

In the model of sepsis induced by CLP, the literature shows that there is translocation of endotoxins and pathogens from the peritoneal cavity to the liver through the portal-hepatic system, triggering the inflammatory process and leading to an increase in cytokines such as TNF-α and IL-1β, as well as ROS and NO [42]. The liver is composed of hepatic sinusoids, made up of subsets of effector system cells such as hepatocytes, Kupffer cells, and polymorphonuclear leukocytes, which contribute to immune surveillance and bacterial clearance [43]. These sites detect and sustain inflammatory responses to antigens, endotoxins, and microorganisms present in the circulation [44]. Thus, the liver represents the second line of defense capable of preventing bacterial translocation from the intestine to the body via the bloodstream. Therefore, liver damage is considered one of the main factors for mortality in sepsis [45].

In our study, an increase in bacterial load was identified 24 h after sepsis induction with an increase in neutrophil infiltration and edema in hepatocytes in the CLP+SAL group, as observed through increased liver mass and MPO levels. On the other hand, the CLP+LF and CLP+LF-ERTA groups did not develop an exacerbated inflammatory response and, consequently, did not have hepatic damage. This result was observed through low levels of ALT, AST, and MDA, as well as a predominant anti-inflammatory profile. A study by [44] shows that immune dysregulation and increased production of free radicals can exhaustively stimulate the cells of the hepatic sinusoids, promoting cell death, tissue damage, and, consequently, reduced bacterial clearance, metabolic disorders, and multiple rgan dysfunctions (MODS). According to [46], the reduction in oxidative stress, inflammatory process, and cell death promoted liver protection during sepsis, demonstrated by the decrease in transaminases (ALT/AST).

**Figure 6:**
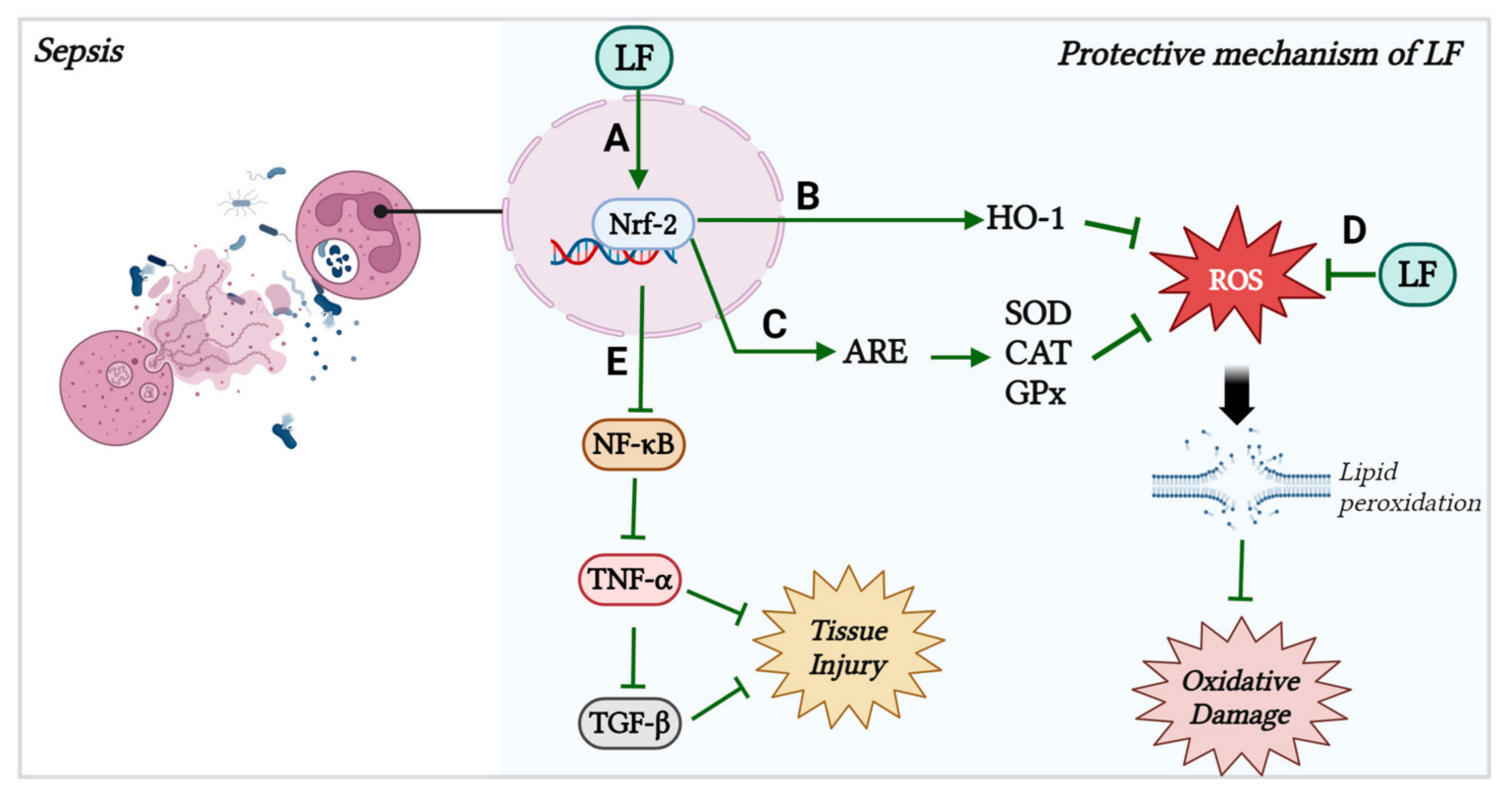
(**A**) After interacting with LF, Nrf-2 is released from its ligand Kelch-like ECH-associated protein 1 (KEAP1) and moves to the nucleus, where it binds to DNA at specific regions to promote the gene expression of the (**B**) enzyme heme oxygenase-1 (HO-1) and (**C**) antioxidant response elements (AREs). This pathway results in an increase in antioxidant defense enzymes such as superoxide dismutase (SOD), catalase (CAT), and glutathione peroxidase (GPx), as well as HO-1 itself. Consequently, ROS levels can be reduced, improving the inflammatory and oxidative stress environment, as occurs in sepsis. (**D**) On the other hand, LF can directly stabilize ROS through mechanisms such as electron donation, hydrogen atom donation, or the formation of adducts between LF and free radicals. The result of reducing free radicals in the intracellular environment is decreased lipid membrane damage due to peroxidation, and reduced redox signaling for pro-inflammatory cytokines. Consequently, oxidative damage is avoided, and the stimulus for inflammation is reduced. (**E**) Another possible pathway where LF may interact involves the stimulation of Nrf-2, which inhibits NF-κB, leading to a reduction in the expression of TNF-α and TGF-β. This reduction mitigates inflammatory mechanisms and cellular and tissue damage. Created with BioRender.com (Agreement number: IU273VUPOD).

The protective mechanism of ergosterol in the development of hepatic dysfunction can further be justified by the increased levels of Nrf-2 [47]. A study by [48] showed that Nrf-2 deficiency in septic mice resulted in liver damage with elevated levels of ALT, AST, and MDA, and increased pro-inflammatory cytokines (TNF-α and IL-1β). As shown in the study by [47], this was due to ergosterol acting as an activator of the Nrf2 factor pathway, which led to the release of Nrf2 from KEAP1 and its subsequent translocation to the nucleus, where it activated the expression of antioxidant genes (through ARE (antioxidant response element)), such as heme oxygenase-1 (HO-1), which acted as a defense mechanism against ROS, reducing oxidative stress and inflammation, and, consequently, tissue damage. (Figure 6)

According to [49], another hepatic protective mechanism of ergosterol is through the positive regulation of the peroxisome proliferator-activated receptor gamma (PPARγ). As shown by [50], the upregulation of PPARγ contributed to the reduction of pyroptosis and liver injury markers such as AST and ALT, as well as decreased oxidative damage during induced sepsis in mice. Furthermore, [50] showed that PPARγ stimulated the activation of Nrf2, promoting a decrease in ROS and, consequently, hepatic oxidative stress.

The structure of PPARs resembles steroid receptors, which are activated by ergosterol. PPARγ activation negatively regulates pro-inflammatory cytokines TNF-α, IFN-γ, IL-2, IL-1β, IL-6, MCP-1, and MIP-1β and reduces TLR-4 activation. In addition, PPARγ contributes to the polarization of macrophages towards the M2 phenotype by regulating M2 target genes, such as Arg-1 [51]. M2 has an anti-inflammatory and immunoregulatory profile [52], promoting tissue repair and resolution of the inflammatory process through the release of mediators such as IL-4, IL-10, and TGF-β [53]. Therefore, the positive regulation of PPARγ by ergosterol can contribute to an anti-inflammatory microenvironment.

Despite these promising results, our study presents some limitations that must be considered. Firstly, the specific dose of LF used was not explored for different dosages and their effects. Moreover, the study duration was restricted to the immediate period following sepsis induction and treatment. Long-term studies are necessary to evaluate the ongoing effects and potential side effects of LF treatment, both alone and in combination with ertapenem. Additionally, the research was conducted on an animal model (*Mus musculus*), which may limit the direct translation of the findings to clinical practice in humans. However, the CLP model is used in several studies and is the gold standard of sepsis models that precedes the application of these results for research with humans [54,55,56]. Therefore, as shown in the present study, the promising results after using LF in a CLP model are the initial proof of the feasibility of applying the lipid fraction in humans, thus justifying its subsequent use in clinical research.

In this regard, in terms of future directions, we suggest that subsequent studies explore the efficacy of LF in sepsis models induced by different pathogens, beyond CLP, to assess the robustness of the therapeutic effects observed. It would also be valuable to investigate the impact of LF in other inflammatory and infectious conditions, broadening the therapeutic potential of this nutraceutical. Conducting clinical trials in humans is essential to confirm the safety and efficacy of LF in the treatment of sepsis. Such studies should consider interindividual variability and potential interactions with other medications commonly used in septic patients. Finally, additional research could focus on developing optimized formulations of LF to maximize its bioavailability and therapeutic efficacy. Exploring combinations of LF with other therapeutic agents, beyond ertapenem, may reveal new synergistic approaches for sepsis treatment. We conclude that, while our study provides initial promising evidence of the therapeutic potential of LF, further research is needed to overcome the mentioned limitations and advance the clinical application of this treatment.

## 5. Conclusions

As expected in this model of lethal sepsis induced by CLP, septic animals exhibited decreased survival and deterioration in clinical parameters such as appearance, responsiveness, temperature, and body weight. On the other hand, the treatment with 0.2 mg·kg^−1^ of LF alone or combined with the antibiotic ertapenem (30 mg·kg^−1^) kept the animals alive during the evaluated period, and an improvement in clinical parameters was observed. These treatments protected the animals from the development of hepatic damage by reducing bacterial load and oxidative stress. In this context, the present study demonstrated that the use of LF as a treatment, as well as in conjunction with antibiotic therapy in sepsis, proved promising due to its antioxidant, antimicrobial, and immunomodulatory activity.

## Data Availability

The raw data supporting the conclusions of this article will be made available by the authors on request.
